# Early administration of romosozumab prevents rebound of bone resorption related to denosumab withdrawal in fractured post-menopausal women: a real-world prospective study

**DOI:** 10.1007/s40618-025-02542-3

**Published:** 2025-01-31

**Authors:** Alberto Piasentier, Alessandro Fanti, Maria Francesca Birtolo, Walter Vena, Roberto Colle, Lucrezia Maria Silvana Gentile, Simona Jaafar, Antonio Carlo Bossi, Andrea G. Lania, Gherardo Mazziotti

**Affiliations:** 1 Department of Biomedical Sciences, Humanitas University, Pieve Emanuele (MI), Via Rita Levi Montalcini 4, Milan, Italy; 2 Endocrinology, Diabetology and Medical Andrology Unit, IRCCS Humanitas Research Hospital, Rozzano, MI Italy; 3 Endocrinology and Diabetes Unit, Humanitas Gavazzeni, Bergamo, Italy

**Keywords:** Romosozumab, Denosumab, Osteoporosis, Fractures, Post-menopausal women, Sequential therapies

## Abstract

**Purpose:**

The real-world effectiveness of switching from denosumab to romosozumab remains controversial. Sequential therapy with romosozumab was shown to be associated with inadequate suppression of bone resorption and there was anecdotal evidence of major osteoporotic fractures (MOFs) after transitioning from denosumab to romosozumab. This study evaluated the effects on bone resorption of early romosozumab administration 3 months after denosumab withdrawal in fractured women with post-menopausal osteoporosis.

**Methods:**

This prospective, single-center cohort study included 39 post-menopausal women with osteoporosis experiencing either MOFs or decrease in bone mineral density during long-term treatment with anti-resorptive drugs. Eighteen received romosozumab either 6 months (Group A) or 3 months (Group B) after their last denosumab dose, while 21 women switched from bisphosphonates to romosozumab and were enrolled as controls (Group C). Serum C-terminal telopeptide of type I collagen (CTX) levels were measured at baseline, 3 and 6 months.

**Results:**

All women of group A and 4 out of 8 women of group B showed a clinically significant increase of CTX values (i.e., change above the least significant change) (*p* = 0.023), which occurred earlier in group A as compared to group B. Moreover, 9/10 women of group A and 2/8 women of group B achieved values above the mean of reference range for pre-menopausal women (*p* = 0.013). In group C, serum CTX values did not change significantly during the follow-up. Two women in Group A experienced MOFs during the follow-up.

**Conclusions:**

Early romosozumab administration after denosumab withdrawal may control bone turnover rebound and possibly prevent incidence of fractures in post-menopausal osteoporosis.

## Introduction

Current therapies approved for post-menopausal osteoporosis include anti-resorptive (i.e., drugs inhibiting osteoclastogenesis and bone resorption) and anabolic drugs (i.e., drugs stimulating osteoblastogenesis and bone formation). Romosozumab is a fully humanized anti-sclerostin-antibody with a dual action on bone remodeling by stimulating osteoblasts and inhibiting osteoclasts, with the potential to address unmet needs in osteoporosis management [1]. Notwithstanding the evidence that bone-builder agents are mostly effective when administered as initial therapy of osteoporosis at very-high risk of fractures [2], in clinical practice anabolic drugs are often commonly used as second-line therapy in patients with intolerance or no response to anti-resorptive drugs, mostly in relation with country-specific policies on drug reimbursement. As a matter of fact, the use of anabolic drugs as second-line therapy after antiresorptives may lead to reduce their effectiveness [3–6]. The classical example is the administration of teriparatide after denosumab that results in a transient decline in lumbar spine and, particularly, in total hip bone mineral density (BMD), reduction in trabecular bone score (TBS), total and cortical volumetric BMD (vBMD) and bone strength with possible increase in fracture risk [4–6]. These effects are closely related to the rebound in bone turnover occurring after denosumab withdrawal and amplified by teriparatide sequential therapy [4–7]. What happens after transitioning from denosumab to romosozumab in subjects with persistently high risk of fractures during denosumab therapy is still a matter of debate [8]. Although an improvement in BMD was reported in subjects treated with romosozumab after short-term denosumab therapy [7, 9, 10], romosozumab might not completely prevent the rebound of bone resorption after denosumab withdrawal [11] and there is anecdotal evidence of incident fragility fractures associated with high bone turnover during treatment of romosozumab post-denosumab [12–14]. In this context, it was proposed either to continue with denosumab in combination with romosozumab [15] or to start romosozumab early after 3 months from the last denosumab dose [12]. This last approach seems to be interesting and effective, but no comparison with the standard sequential approach (i.e., commencing romosozumab 6 months after the last dose of denosumab) in a prospective way has been so far published.

In this prospective study, we evaluated whether the overlapping strategy of transition from denosumab to romosozumab may provide an advantage in terms of controlled rebound of bone resorption over the standard approach in post-menopausal women who had experienced either major osteoporotic fracture (MOFs) or decrease in BMD during denosumab therapy.

## Materials and methods

This is a prospective, single-center, real-world cohort study, conducted at the Endocrinology Unit of IRCCS Humanitas Research Hospital (Rozzano-Milan, Italy). The study followed the Strengthening the Reporting of Observational Studies in Epidemiology (STROBE) reporting guidelines [16]. The inclusion criteria were: (1) post-menopausal women with documented osteoporosis and 10-year MOF risk ≥ 20% (determined with DeFRA tool, a validated fracture risk assessment tool derived from FRAX plus) [17]; (2) T-score at the spine or femur of less than − 2.5 standard deviation (SD) (or less than − 2.0 if there were ≥ 2 moderate or severe vertebral fractures or if there was a femoral fracture in the previous 2 years); (3) history of ≥ 1 moderate or severe vertebral fractures or ≥ 2 mild vertebral fractures or ≥ 1 femoral fracture or history of ≥ 2 non-vertebral non-femoral MOFs); (4) incident MOF and/or decrease in BMD over the least significant change (LSC) thresholds at any skeletal site [18] as evaluated by the same DXA machine after at least one year of treatment; (5) written informed consent. All women treated with denosumab had optimal medication adherence as defined by regular administration of the drug every 6 months ± 4 weeks [19]; 6). Exclusion criteria were: (1) history of previous myocardial infarction or stroke; (2) secondary osteoporosis; (3) history of bone diseases other than osteoporosis (e.g., Paget’s disease of the bone); (4) history of malignancy of the bone; (5) severe liver or kidney disease (estimated glomerular filtration rate (eGFR) *<* 30 mL/min or Child–Pugh grade B or C). Among patients regularly followed-up at the Endocrinology Unit of IRCCS Humanitas Research Hospital in the period between September 2022 and October 2024, 39 women (median age 67 years, range: 52–85) met the inclusion and exclusion criteria and were enrolled in the study. The median duration of anti-resorptive therapy was 36 months (range: 12–96). Eighteen women previously treated with denosumab were prescribed sequential therapy with romosozumab by two protocols according to the timing of admission at the outpatient bone clinic: 10 women started romosozumab 5–6 months after the last denosumab administration (**Group A**), whereas 8 women started romosozumab 3–4 months after the last denosumab dose (**Group B**). Twenty-one consecutive women transitioning from bisphosphonates to romosozumab were enrolled as controls (**Group C**), in order to minimize the type II error correlated to large range variability of bone turnover markers [20] and small size of study groups.

Since the rapid increase in bone resorption has been consistently associated with bone loss and fractures after denosumab withdrawal [21], the first endpoint of the study was the changes in C-terminal telopeptide of type I collagen (CTX, i.e. a marker of bone resorption) during 6 months of follow-up after starting romosozumab. As exploratory endpoints we evaluated (1) the number of patients achieving CTX values above the mean of reference range for pre-menopausal women [22]; (2) occurrence of clinical fractures during the follow-up; (3) effects of zoledronate on serum CTX when administered in combination with romosozumab after denosumab withdrawal.

All women were evaluated for serum CTX at baseline (before romosozumab administration) and after 3 and 6 months of follow-up. Moreover, clinical fractures were registered during the follow-up. According to the clinical judgement, a single administration of Zoledronate 5 mg i.v. was performed in combination with romosozumab in women showing clinically significant increase of CTX with or without incident fractures.

The protocol was approved by the Ethics Committee of IRCCS Humanitas Research Hospital and the enrolled women gave informed consent for the study.

### DXA measurement of BMD

Before romosozumab prescription, during treatment with anti-resorptive drugs, women had been evaluated by DXA measurement of BMD at lumbar spine, femoral neck and total hip every 18–24 months. The same machine was used for the longitudinal evaluations of BMD. BMD was expressed as gr/cm^2^ and T-score, comparing the results with those obtained in a gender-matched Caucasian population at the peak of bone mass [18]. A T-score less than or equal to − 2.5 SD at the hip or spine was defined as osteoporosis, whereas osteopenia was defined as a T-score between − 1 and − 2.5 SD. Due to the short-term follow-up, it was not permitted by the national health agency to prescribe a second DXA during romosozumab treatment.

## Biochemical assessments

CTX was measured in blood samples on the morning after an overnight fasting, using the Elecsys β-CrossLaps/serum assay based on electrochemiluminescence technology and the COBAS e801 immunoanalyzer. The intra-individual coefficient of variation was 9.4% (4.1–27%) with a LSC of 27%. The reference ranges for pre- and post-menopausal women were 0.136–0.689 and 0.177–1.015 ng/ml, respectively. The mean of reference range of pre-menopausal women was 0.306 ng/ml. As previously established [21, 22], CTX values above this cut-off were considered as predictor of bone loss after denosumab withdrawal.

### Statistical analysis

Continuous and categorical data were presented as mean and 95% confidence interval (95% C.I.) of the mean, and as number and percentage, respectively. Unpaired and paired data were compared using Wilcoxon’s, Mann–Whitney’s and Kruskal-Wallis’ tests, respectively. Unpaired frequencies were compared using the Chi-square test. A *p* < 0.05 was considered as significant.

## Results

At study entry, women of groups A and B already treated with denosumab showed significantly (*p* = 0.002) lower values of CTX as compared to control women already treated with bisphosphonates, without significant differences in age (*p* = 0.372), duration of anti-resorptive therapies (*p* = 0.772), BMD values at lumbar spine (*p* = 0.963), femoral neck (0.926) and total hip (*p* = 0.825) (Table 1). Most of women (32/39) transitioned from anti-resorptive drugs to romosozumab due to incident MOFs, without significant difference among the three study groups (Table 1). In all these fractured women, BMD had not been increased during anti-resorptive therapy. In only 7 women (17.9%), the shift from anti-resorptive drugs to romosozumab was motivated by decrease in BMD values in at least two skeletal sites during the treatments (Table 1).

**Table 1:** Clinical data of post-menopausal women starting romosozumab after denosumab (groups A, B) or bisphosphonates (group C). In group A, romosozumab was started 5–6 months after the last denosumab administration, whereas in group B the first dose of romosozumab was administered earlier 3–4 months after last dose of denosumab. Continuous data were presented as mean and 95% confidence interval of the mean, whereas categorical data were presented as number and frequencies. Comparisons were performed by non-parametric tests.*, *p* < 0.01 vs. groups A and B

	Group A	Group B	Group C	*p*-values
Cases (N.)	10	8	21	
Age (years)	64 (58–70)	72 (63–81)	67 (61–72)	0.372
Duration of previous anti-resorptive therapy (months)	40 (20–59)	47 (24–69)	42 (30–55)	0.772
Recent MOFs (n./%) VFs Hip Fx NVFs	7 (70.0%) 5 (50.0%) 0 2 (20.0%)	8 (100%) 7 (87.5%) 0 1 (12.5%)	17 (81.0%) 12 (57.1%) 1 (4.8%) 4 (19.0%)	0.252
Last LS BMD T-score (SD) before romosozumab Tx	−2.92 (−3.68 to −2.15)	−2.91 (−3.73 to −2.08)	−2.90 (−3.35 to-2.52)	0.963
Last FN BMD T-score (SD) before romosozumab Tx	−2.28 (−3.30 to −2.05)	−2.25 (−4.20 to −0.31)	−2.84 (−3.21 to-2.47)	0.926
Last TH BMD T-score (SD) before romosozumab Tx	−2.64 (−3.37 to −1.91)	−2.65 (−3.12 to −2.13)	−2.53 (−2.89 to-2.16)	0.825
Decrease in BMD during anti-resorptive therapies (n./%)	3 (30.0%)	0	4 (19.0%)	0.252
Baseline serum CTX values (ng/ml)	0.09 (0.06–0.12)	0.07 (0.03–0.19)	0.20 (0.14–0.26)*	0.002

BMD, bone mineral density; CTX, C-terminal telopeptide of type I collagen; FN, femoral neck; Fx, fractures; LS, lumbar spine; MOFs, major osteoporotic fractures; NVFs, non-vertebral fractures; SD, standard deviation; TH, total hip; Tx, therapy; VFs, vertebral fractures

**Fig. 1:**
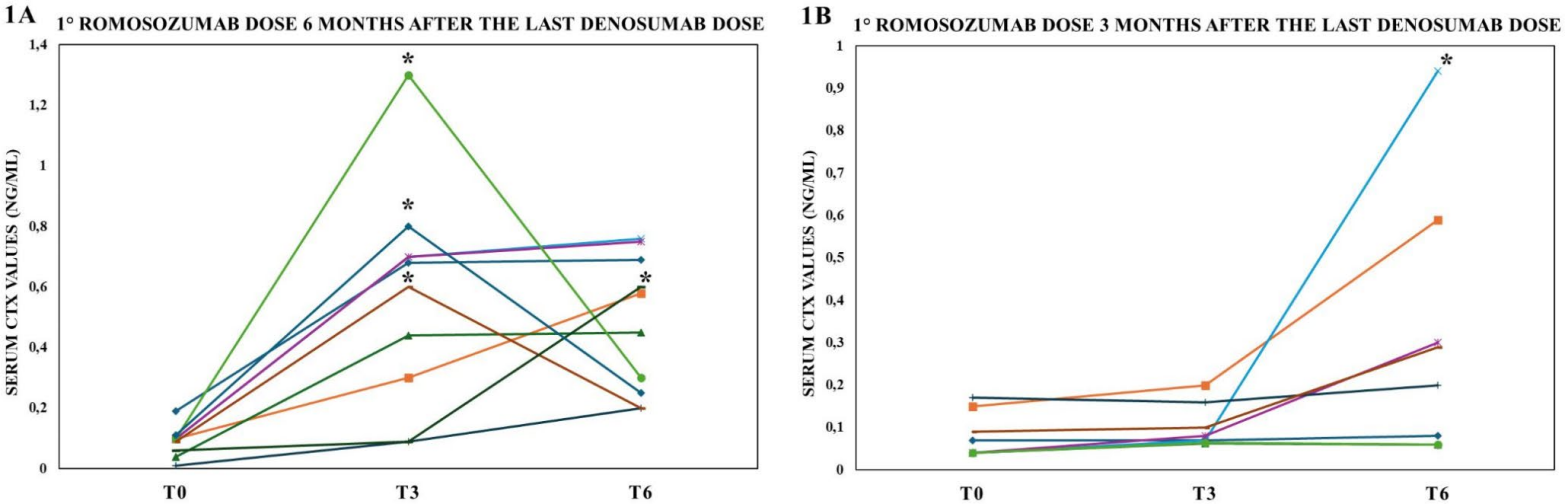
Changes in serum C-terminal telopeptide of type I collagen (CTX) values during the first 6 months of romosozumab therapy which was started either 5–6 months (1a) or 3–4 months (1b) after the last dose of denosumab. Comparisons were performed by non-parametric tests. *, i.v. administration of zoledronate 5 mg was performed **T0**: baseline evaluation; **T3**: three months follow-up; **T6**: six months follow-up

During the follow-up, serum CTX values increased significantly in both groups in whom subjects had been already treated with denosumab, although the increase occurred earlier in group A as compared to group B (Fig. 1a, b). All women of group A and 4 out of 8 of women of group B showed an increase in CTX values of above the LSC (*p* = 0.023). Moreover, 9/10 women of group A e 2/8 women of group B achieved CTX values above the mean value of reference range for pre-menopausal women (*p* = 0.013). Two women of Group A experienced incident MOFs (vertebral and distal radius fractures) during the first 3 months of romosozumab sequential therapy. In these women, an administration of zoledronate 5 mg i.v. was performed in combination with romosozumab and serum CTX returned to the baseline values and no other fractures occurred during the follow-up (Fig. 1a). Zoledronate was also administered at different time-points in other 3 women who had reported clinically relevant increase in serum CTX values during the follow-up (Fig. 1a, b). In control women of group C already treated with bisphosphonates, serum CTX values did not change significantly during the 6-month follow-up after starting romosozumab (from 0.20 ng/ml, 95% C.I. 0.13–0.26 to 0.16 ng/ml, 95% C.I. 0.12–0.20; *p* = 0.277).

## Discussion

In post-menopausal women experiencing MOFs or bone loss during denosumab therapy, the standard sequential therapy with romosozumab (i.e., the first dose of romosozumab administered 6 months after last dose of denosumab) did not control the rebound in bone resorption related to denosumab withdrawal and some women experienced new fractures. However, when the first dose of romosozumab was anticipated three months after the last denosumab dose, rebound in bone resorption was blunted in majority of patients. This study also suggests that administration of zoledronate in combination with romosozumab may provide an advantage in controlling the rebound in bone turnover with rapid decrease in serum CTX values.

During the last years, several observational studies have evaluated the effectiveness of romosozumab in post-menopausal women already treated with either anti-resorptive drugs or teriparatide [23]. In this prospective study, we evaluated the real-world effectiveness of romosozumab in the specific setting of post-menopausal women experiencing new MOFs or decrease in BMD during treatment with denosumab [24]. Noteworthy, the fractures were not caused by a delay in denosumab administration [19], since the medication adherence was optimal in all the enrolled women. However, it is uncertain whether these women might be defined as “non responder” to denosumab, since the available evidence does not permit a firm assessment of the success or failure of treatments in osteoporosis [24]. On the other hand, reimbursement agencies and health technology assessments categorize first- and second-line drugs and clinicians are persuaded to change the therapeutic strategy in subjects experiencing fractures during the course of treatment, although they cannot be taken as proof of treatment failure [24]. As a matter of fact, the shift from denosumab to other bone-active drugs may be a challenge, since the drug withdrawal is associated with rebound in bone turnover associated with a further increase in fracture risk [21, 25]. Zoledronate and alendronate could control the rebound of bone resorption with prevention of bone loss [26], but the effectiveness bisphosphonates is lower than denosumab [27, 28] making not fully appropriate their use as second-line therapy in women experiencing fractures during denosumab. In this clinical setting, romosozumab may be a valuable option thanks to its higher effectiveness than denosumab in increasing BMD [29], improving TBS [30, 31] and decreasing risk of vertebral fractures [32]. Moreover, romosozumab was shown to be effective and safe when used immediately after a MOFs to prevent the so called “imminent risk” of subsequent fractures [33]. All these data provided a strong rationale for transitioning from denosumab to romosozumab in our women, although the anti-resorptive effects of romosozumab seem to be blunted when the drug is used as second-line therapy after anti-resorptive drugs [10, 34, 35] and there was anecdotal reports of progression of fractures when romosozumab was used after denosumab therapy [12–14].

Our study focused on evaluating the effects of second-line therapy with romosozumab on bone resorption. Serum CTX values are currently considered the best biochemical marker guiding the therapeutic decision-making after denosumab withdrawal [21, 36], since there is a close relationship between the increase in serum CTX values and the entity of bone loss after denosumab withdrawal [22, 37]. In our study, all women starting romosozumab according to the standard protocol (i.e., 6 months after the last denosumab dose) showed clinically relevant increase of serum CTX values and in most of these cases the values resulted to be above the low reference range for pre-menopausal women, that is considered a safe target for preventing bone loss after denosumab discontinuation [21, 22]. Consistently, some of our women with very high CTX values experienced MOFs during the first 2–3 months after starting romosozumab therapy. An original aspect of our study was the comparison between the standard protocol of sequential therapy and a novel approach recently proposed by a clinical case series [12] that consisted in giving the first dose of romosozumab 3 months after the last dose of denosumab. This latter approach appeared to be effective in preventing the clinically significant increase in serum CTX values in one-half of women, whereas in the remaining cases the increase of CTX values was smaller and later than that observed in women starting romosozumab 6 months after the last denosumab dose. Moreover, only 25% of women receiving romosozumab 3 months after denosumab showed CTX values above the safe reference range for pre-menopausal women [22]. All these findings would suggest that women starting early romosozumab might be at lower risk of bone loss and fractures as compared to the women undergoing standard sequential therapy. Moreover, our study also suggests that monitoring of CTX values should be performed in patients undergoing treatment with romosozumab after denosumab in order to early identify the cases predisposed to bone loss, such as already indicated for patients transitioning from denosumab to bisphosphonates [21].

Combination therapies have been proposed for patients with severe osteoporosis. Specifically, adding teriparatide to denosumab was found to be more effective than monotherapy [4–6] and this approach has been recommended for patients experiencing fractures during denosumab therapy [21]. More recently, alternative approaches have been proposed consisting in prescribing romosozumab in a variable degree of combination with ongoing denosumab therapy, based on the concept that romosozumab might stimulate bone formation even in patients with a closed resorption phase, as evidenced by long-term denosumab treatment and markedly suppressed bone turnover [12, 15]. In our study, we performed in few cases a new combination therapy which consisted in addition of zoledronate to the ongoing romosozumab therapy for those women showing clinically significant increase in serum CTX with or without incident MOFs. This approach appeared to be effective since CTX values decreased significantly after zoledronate administration at least during only 3 months of follow-up. Further studies will clarify whether this approach may be effective in the long-term and whether it might offer the opportunity to use teriparatide after romosozumab therapy.

This study has limitations. The short-term follow-up did not allow to perform a densitometric comparison between the two sequential protocols of transitioning from denosumab to romosozumab. The number of enrolled patients was low in relationship with the monocentric nature of the study and the low number of patients experiencing fractures or bone loss during treatment with denosumab [28, 38]. Indeed, one could argue that serum bone turnover markers have a large range of variability [20] and the study was underpowered to obtain reliable clinical indications. However, the study protocol considered as end-points the CTX changes higher than the LSC [39] and the achievement of CTX values above the mean values of range for pre-menopausal women [21] that were related to bone loss after denosumab withdrawal [22, 36, 37]. Moreover, we included a relatively large group of control subjects previously exposed to bisphosphonates in whom bone resorption markers were expected to not change during romososumab therapy [34], in order to better understand that the changes in CTX values observed after denosumab therapy were related to the drug-withdrawal rather than individual variability. For this purpose, a control group of naïve women treated with romosozumab as first-line therapy may have provided more reliable information about the effects of the drug on bone turnover markers [40, 41], but in our clinical practice romosozumab is reimbursed by the national agency of health only as second-line therapy in post-menopausal osteoporosis.

This prospective real-world study suggests that early administration of romosozumab after the last dose of denosumab may prevent the rebound of bone turnover in women with post-menopausal osteoporosis at high risk of fractures.
